# Mechanism of curaxin-dependent nucleosome unfolding by FACT

**DOI:** 10.3389/fmolb.2022.1048117

**Published:** 2022-11-22

**Authors:** Olesya I. Volokh, Anastasia L. Sivkina, Andrey V. Moiseenko, Anna V. Popinako, Maria G. Karlova, Maria E. Valieva, Elena Y. Kotova, Mikhail P. Kirpichnikov, Timothy Formosa, Vasily M. Studitsky, Olga S. Sokolova

**Affiliations:** ^1^ Biology Faculty Lomonosov Moscow State University, Moscow, Russia; ^2^ Semenov Federal Research Center of Chemical Physics RAS, Moscow, Russia; ^3^ Bach Institute of Biochemistry Research Center of Biotechnology of the Russian Academy of Sciences, Moscow, Russia; ^4^ RG Development & Disease Max Planck Institute for Molecular Genetics, Berlin, Germany; ^5^ Institute for Medical and Human Genetics Charité-Universitätsmedizin Berlin, Berlin, Germany; ^6^ Fox Chase Cancer Center, Philadelphia, PA, United States; ^7^ Department of Biochemistry, University of Utah School of Medicine, Salt Lake City, UT, United States

**Keywords:** SPT16, FACT, SSRP1, nucleosome, transmission electron microscopy, SpFRET microscopy

## Abstract

Human FACT (FACT) is a multifunctional histone chaperone involved in transcription, replication and DNA repair. Curaxins are anticancer compounds that induce FACT-dependent nucleosome unfolding and trapping of FACT in the chromatin of cancer cells (c-trapping) through an unknown molecular mechanism. Here, we analyzed the effects of curaxin CBL0137 on nucleosome unfolding by FACT using spFRET and electron microscopy. By itself, FACT adopted multiple conformations, including a novel, compact, four-domain state in which the previously unresolved NTD of the SPT16 subunit of FACT was localized, apparently stabilizing a compact configuration. Multiple, primarily open conformations of FACT-nucleosome complexes were observed during curaxin-supported nucleosome unfolding. The obtained models of intermediates suggest “decision points” in the unfolding/folding pathway where FACT can either promote disassembly or assembly of nucleosomes, with the outcome possibly being influenced by additional factors. The data suggest novel mechanisms of nucleosome unfolding by FACT and c-trapping by curaxins.

## 1 Introduction

The eukaryotic genome is organized into nucleosomes (Luger et al., 1997; Vasudevan et al., 2010) that block DNA accessibility to various sequence-specific DNA-binding proteins. The DNA accessibility is tightly regulated by numerous factors, including ATP-dependent remodelers and ATP-independent histone chaperones (Clapier et al., 2017; Gurova et al., 2018; Formosa and Winston, 2020; Zhou et al., 2020). FACT (facilitates chromatin transcription) is a multifunctional histone chaperone, that is, involved in both nucleosome assembly and a large-scale, ATP-independent, reversible nucleosome unfolding that increases DNA accessibility to various factors (Valieva et al., 2016; Valieva et al., 2017; Sivkina et al., 2022). These activities of FACT contribute to various nuclear processes including transcription, replication and repair (Xin et al., 2009; Valieva et al., 2016; Gurova et al., 2018).

**FIGURE 1:**
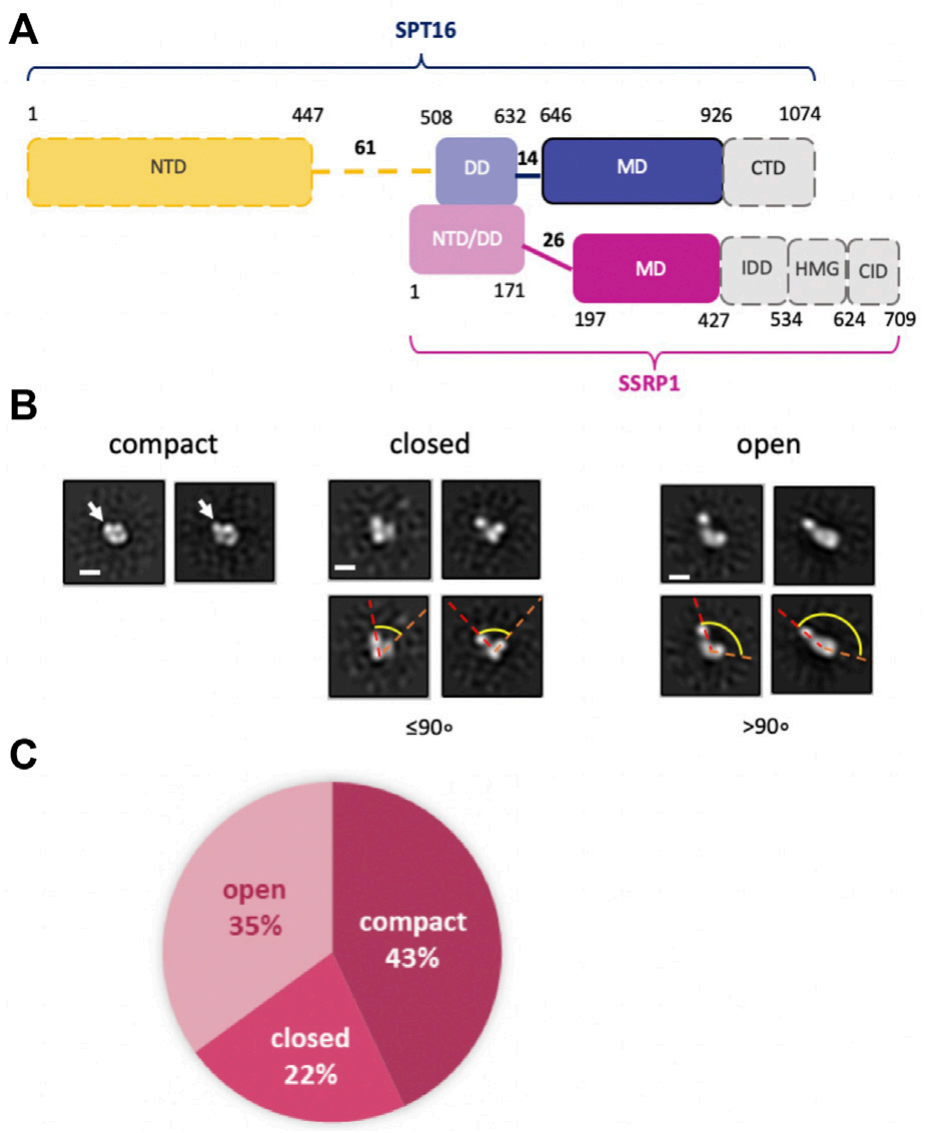
Human FACT is a flexible protein complex. **(A)** Human FACT domain architecture. Gray domains with dashed boundaries are expected to be disordered and were not detected by cryo-EM (). Domain abbreviations: NTD—N-terminal, DD—dimerization, MD—middle, CTD—C-terminal, HMG—high mobility group box, IDD—intrinsically disordered, CID—CTD-Interacting domain, **(B)** Characteristic 2D class averages of FACT in compact, closed and open conformations: the arrow indicates the domain, that is, detectable only in the compact conformation, dashed lines highlight the angle between the domains, which increases as the complex opens. **(C)** Distribution (%) of FACT in different conformations. Bar—10 nm.

FACT is a conserved protein complex (Gurova et al., 2018) that consists of two subunits: SPT16 (Suppressor of Ty 16) and SSRP1 (Structure Specific Recognition Protein 1) in human and plants; Pob3 (Polymerase One Binding protein 3) replaces SSRP1 in yeasts. Human SPT16 and SSRP1 subunits consist of four and five structurally different regions, respectively (Figure 1A), implicated in binding to both nucleosomal DNA and to different core histones [see (Clapier et al., 2017; Gurova et al., 2018; Formosa and Winston, 2020; Zhou et al., 2020) for review].

FACT inhibition negatively affects a number of the critical p53-, NF-kB- and HSF1-dependent metabolic pathways involved in cancer development and leads to the death of the cancer cells (Gasparian et al., 2011). Human FACT (FACT) is overexpressed in various types of cancer and thus is a promising target for anti-cancer drugs (Gasparian et al., 2011; Garcia et al., 2013; Fleyshman et al., 2017). In particular, members of the class of DNA intercalators called curaxins have strong anticancer activity (Gasparian et al., 2011; Dermawan et al., 2016), induce chromatin trapping (c-trapping) of FACT and strongly inhibit normal human FACT activities *in vivo* (Gasparian et al., 2011). C-trapping of FACT involves formation of Z-DNA *in cellulo* (Safina et al., 2017) and curaxin-dependent nucleosome unfolding accompanied by tight binding of FACT to the unfolded nucleosomes *in vitro* (Chang et al., 2018). The anticancer activity of curaxins is highly dependent on c-trapping of FACT (Gasparian et al., 2011; Safina et al., 2017; Chang et al., 2018).

Recently, using transmission electron microscopy we have described a number of topologically different structures of yeast FACT(yFACT)-nucleosome complexes formed during nucleosome unfolding by yFACT and Nhp6 protein (Sivkina et al., 2022). Our data suggested that recently determined high resolution EM 3D maps of human FACT bound to subnucleosomal complexes (Mayanagi et al., 2019; Liu et al., 2020) are structurally similar to the early intermediates formed during nucleosome unfolding. It was also shown that in the unfolded complexes yFACT is engaged in multiple interactions, both with nucleosomal DNA and core histones (Sivkina et al., 2022), raising the possibility that a similar mechanism could be involved in curaxin-dependent nucleosome unfolding by human FACT.

Here spFRET microscopy, single particle transmission electron microscopy (TEM) and molecular modeling (MM) revealed high conformational flexibility of both FACT and FACT-nucleosome complexes formed in the presence of the clinically relevant curaxin CBL0137. The structural organization of the intermediates formed during the FACT/curaxin-dependent nucleosome unfolding illuminate the mechanism of this process and the mechanism of c-trapping of FACT by curaxins.

## 2 Results

### 2.1 Human FACT is a flexible protein complex

The spatial organization of human FACT (FACT, Figure 1A) was studied using transmission electron microscopy (TEM). To ensure correct alignment of the particles, they were classified in RELION2.1 (Supplementary Table S1 and Supplementary Figure S1). TEM revealed three distinct conformations of FACT: compact, closed and open (Figure 1B). The closed conformation includes three closely positioned densities that dissociate from one another in the open conformation (Figure 1B). These complexes are structurally similar to those detected previously during TEM studies of yeast FACT (Sivkina et al., 2022). Here TEM also revealed a novel, abundant, compact conformation of FACT that consists of four globular densities arranged in a compact diamond-like shape (Figure 1B) that was not detected previously with yeast FACT (Sivkina et al., 2022). The three conformations were present at ratios of approximately 2:1:1.6 (compact:closed:open) (Figure 1C).

Based on the previous identification of the components of yFACT (Sivkina et al., 2022), the three densities present in all conformations of FACT were tentatively identified as SSRP1-NTD/DD-SPT16-DD, SSRP1-MD, and SPT16-MD (Figure 1A). Accordingly, the 2D projections of those densities are ∼4–5 nm in diameter, as expected for these domains with molecular masses of ∼30–40 kDa. The fourth electron density, detectable only in the compact conformation, is somewhat larger (∼5–6 nm in diameter, Figure 1B), and is therefore likely to be the Spt16-NTD domain, which at ∼50 kDa is the largest domain of FACT (Stuwe et al., 2008). This domain was not detected in previous EM studies of FACT and FACT-nucleosome complexes (Liu et al., 2020; Sivkina et al., 2022), presumably indicating larger conformational flexibility of this region in the EM reconstructions detected.

To further evaluate whether the additional density is indeed the NTD of SPT16, we analyzed a truncated version of FACT lacking this domain (SPT16∆NTD) by TEM. With this construct, only closed (48%) and open (52%) conformations were identified, with no 2D class corresponding to the compact 4-lobed 3D map being observed (Figure 3A). The data are consistent with the proposal that the largest electron density detected in the compact conformation of FACT (Figure 1B) is indeed the SPT16-NTD domain.

### 2.2 FACT domain identification

**FIGURE 2:**
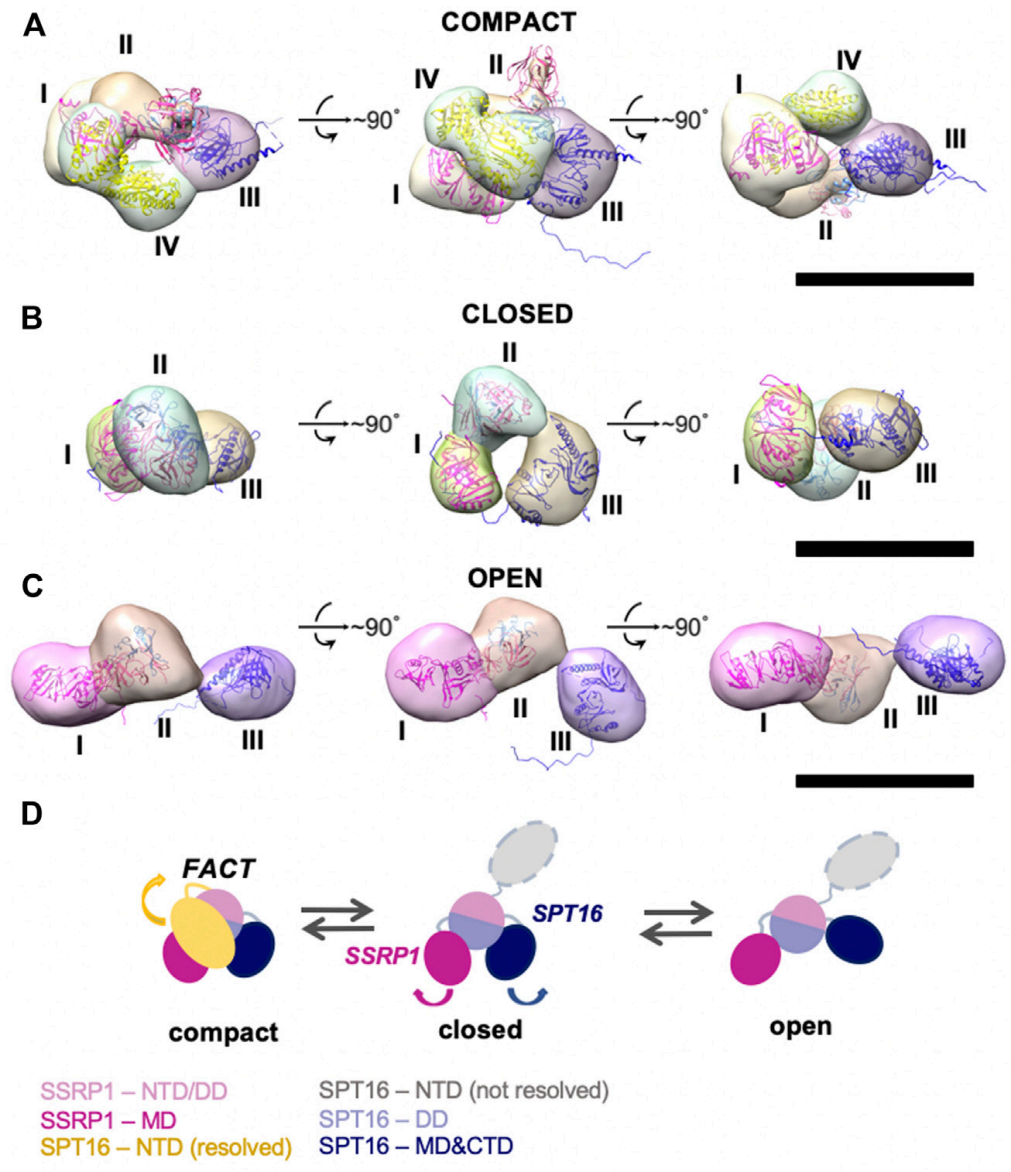
Three-dimensional reconstructions of FACT in compact, closed and open conformations. **(A**–**C)** 3D maps of FACT in the compact **(A)**, closed **(B)** and open **(C)** conformations. In the compact complex, the crystal structures of the domains were docked into corresponding EM densities I-IV with correlation coefficients of 0.92; 0.91; 0.89; 0.93, respectively. Bar—10 nm **(D)**. The proposed pathway of FACT unfolding. The color code for the domains of FACT is shown at the bottom of the figure.

To identify FACT domains and to ensure that the observed conformational states do not simply reflect different orientations of the same configuration, 3D maps of FACT in the compact and open conformations, and in the closed conformation of FACT containing SPT16ΔNTD that produced more homogeneous and better resolved complexes (Figures 2A–C). The resolutions of the reconstructions were moderate (21Å for compact, 34Å for closed and 31Å for open conformations, respectively), reflecting high flexibility of the FACT molecule. The linear dimensions of FACT are 12 ± 0.4 × 8.6 ± 0.7 nm for the compact, 9.1 ± 0.4 × 5.4 ± 0.5 nm for the closed and 15 ± 1.9 × 5.5 ± 0.6 nm for the open conformations, respectively.

To localize the domains in the electron densities of FACT, rigid fitting was performed using the available crystal structures of FACT domains (Figure 2). Based on previous EM studies (Liu et al., 2020; Sivkina et al., 2022), we assumed that the middle density is SSRP1-NTD/DD-SPT16-DD (domain II), while the SSRP1-MD (domain I) and SPT16-MD (domain III) flank it on either side (Figure 2). This structural assignment results in a good fit of all domain structures into the electron densities of the best resolved compact conformation of FACT (Figure 2A). For the closed and open FACT conformations the MD domains of SSRP1 and SPT16 were positioned into the densities I and III based on the length of the linkers connecting the domains. Because the linker connecting NTD/DD and MD domains of SSRP1 is longer than the one connecting NTD/DD and MD of SPT16 (Figure 1A), SSRP1-MD (domain I) is likely to be connected with the NTD/DD through a less extensive electron density than SPT16-MD (Figures 2B,C). Crystal structures were automatically fitted into corresponding domains with correlation coefficients >0.89.

**FIGURE 3:**
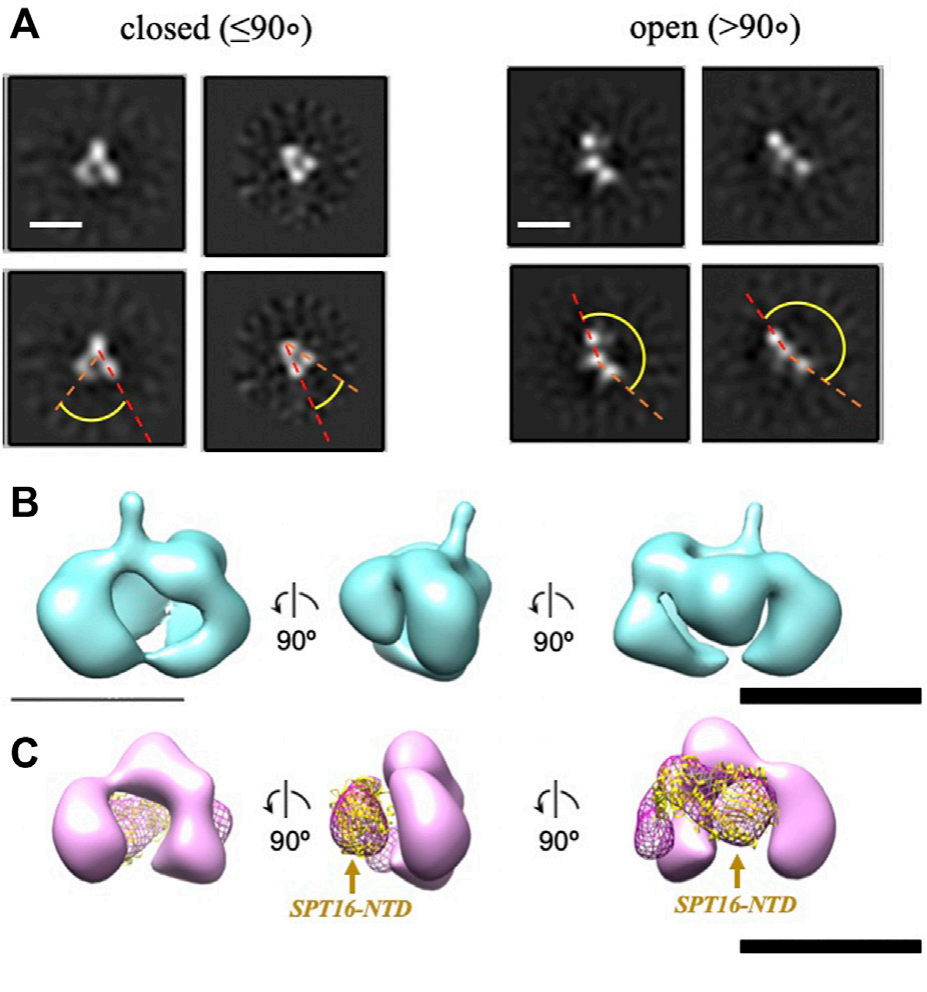
Three-dimensional reconstruction of FACT-SPT16ΔNTD truncated mutant. **(A)** Representative 2D class-averages of the FACT-ΔNTD in the closed and open conformations. Scale bars—10 nm. **(B)** 3D map of FACT in the compact conformation. **(C)** 3D map of the closed conformation of FACT-ΔNTD mutant (pink, the same orientations as in part **(B)** and the difference map with the intact FACT (magenta mesh). Atomic model of SPT16-NTD (yellow ribbon) was docked into the difference map with the correlation coefficient 0.92. Bar—10 nm.

To localize the SPT16-NTD domain, the 3D map of compact conformation of full-length FACT (Figure 3B) was first aligned with the map of closed conformation of the FACT SPT16ΔNTD mutant (Figure 3C). The difference map revealed an additional density in FACT in comparison with the mutant version of the complex (shown in magenta mesh in Figure 3C **)**. Rigid fitting of the crystal structure of SPT16-NTD (pdb ID 5e5b (Marciano and Huang, 2016)) into this density yielded good correspondence of the structures, with correlation coefficient 0.92 (Figure 3C).

**FIGURE 4:**
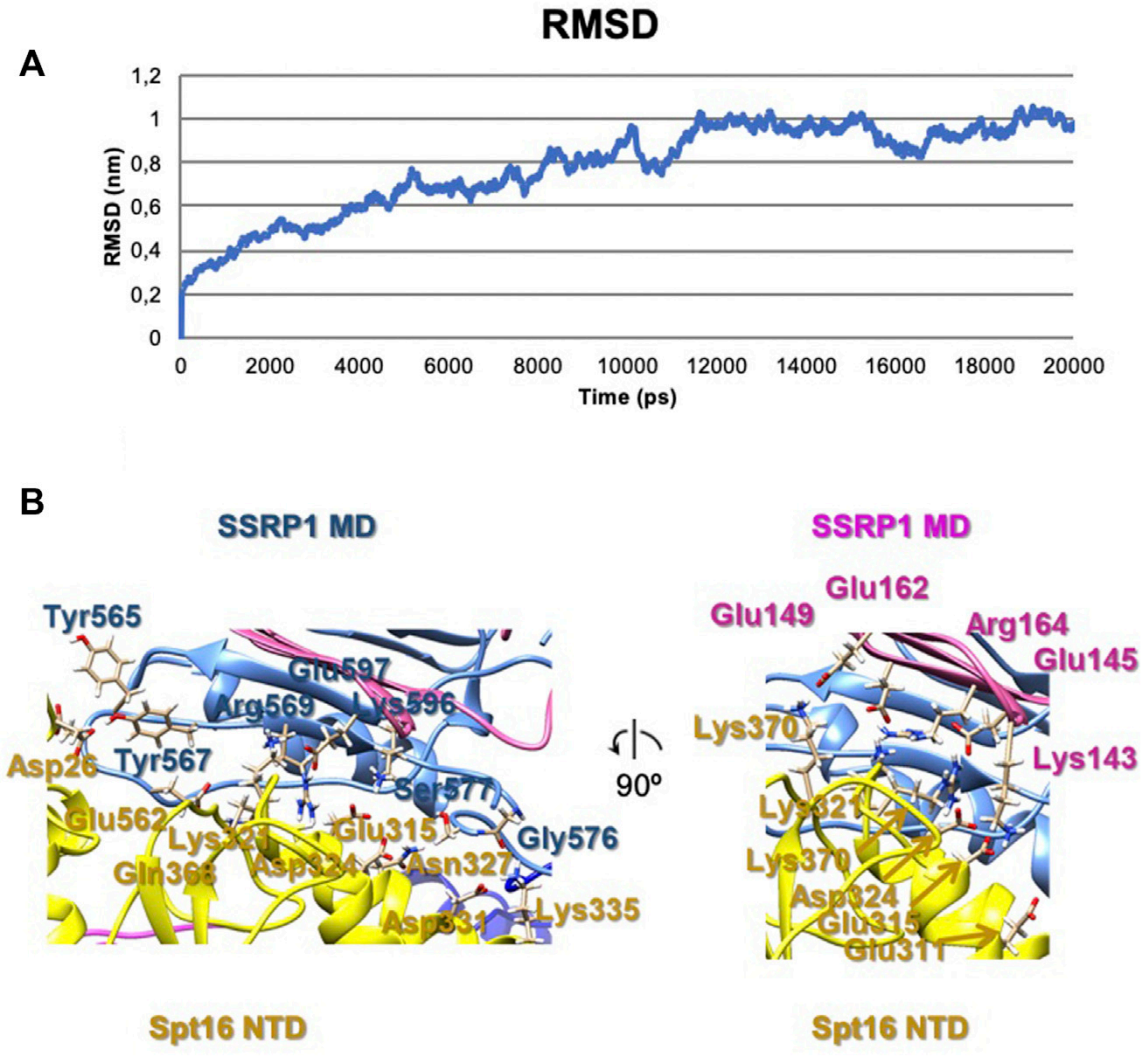
The results of molecular dynamics simulations of the compact FACT conformation. **(A)** RMSD of Cα of the whole complex [(SSRP1-NTD/DD-SPT16-DD (domain II), SSRP1-MD (domain I) and SPT16-MD (domain III)] **(B)** Contacts between the subunits in the complex after molecular dynamics simulations.

To evaluate possible driving forces allowing formation of the compact FACT conformation (Figure 2A), the structural organization of the complex was analyzed using HADDOCK (to determine the initial orientations of the domains, Supplementary Figure S2) and molecular dynamics simulations (for the analyzing of interacting subunits, Figure 4). The trajectories of molecular dynamics simulations allow analysis of the structure and conformational dynamics of the complex at atomic level. The structural changes of the compact FACT conformation were monitored through the evolution of root mean square deviation (RMSD) of Cα atom positions and are shown in Figure 4A. RMSD was calculated for the whole complex (SSRP1-NTD/DD-SPT16-DD (domain II), SSRP1-MD (domain I) and SPT16-MD (domain III)). In the course of the simulations, the RMSD of the whole protein increases during the first nanoseconds of the simulations and then fluctuates within the range of 0.8–1 nm (Figure 4A). The RMSD has a non-diverging trend at the end of the simulations, thus, confirming that the system has reached its equilibrium state.

The correlation coefficient of the model after molecular dynamics with EM-maps was 0.88. As a result, the interactions between SPT16-NTD and other previously resolved domains in the compact conformation of FACT were suggested in this model (Figure 4B). In the model after HADDOCK, three domains of FACT (SSRP1-NTD/DD, SPT16-DD, and SPT16-NTD) are tethered together through hydrophobic interactions between the subunits with the buried surface contact area of ∼2004 Å2 (Supplementary Figure S1). Thus, the hydrophobic interactions that connect different domains in the compact FACT complex are quite strong and are potentially able to stabilize FACT in a compact conformation in solution. The hydrophobic interactions are supplemented by a dense network of hydrogen bonds between the domains in the model of compact FACT conformation after molecular dynamics (some are presented on Figure 4, for complete list of hydrogen bonds see Supplementary Table S2).

Since no open complexes with four densities were observed, the data suggest that SPT16-NTD domain “locks” the other domains of FACT in the compact conformation (Figure 2A), primarily through hydrophobic interactions supplemented by multiple hydrogen bonds (Figure 4). When the NTD is displaced, the remaining domains represent a more flexible elements, that is, in equilibrium between the open and closed states (Figure 2D).

The SPT16-NTD domain is not detectable in the other closed conformations or in any of the open forms, most likely because once it dissociates from the remaining FACT complex it becomes more mobile and therefore “invisible” in the 2D and 3D class averages; indeed, it was not detected in previous structural studies of FACT-nucleosome complexes (Liu et al., 2020). Alternatively, separation of any density from the compact complex could induce separation and mobilization of the SPT16-NTD domain of FACT. This possibility is unlikely because it predicts that all complexes with three densities would be in an open state, but we also observed closed three-density complexes (Figure 1B).

In summary, TEM revealed that FACT is a mixture of three conformations: compact, closed and open. Four or three distinct densities are visible in the compact and closed/open conformations, respectively. The three densities were identified as SSRP1-MD (domain I), SSRP1-NTD/DD-SPT16-DD (domain II) and SPT16-MD (domain III); the fourth domain is SPT16-NTD. The arrangement of the densities in the complexes suggests that SPT16-NTD domain “locks” the other domains of FACT in the compact conformation.

### 2.3 Nucleosome unfolding by FACT in the presence of curaxin CBL0137

**FIGURE 5:**
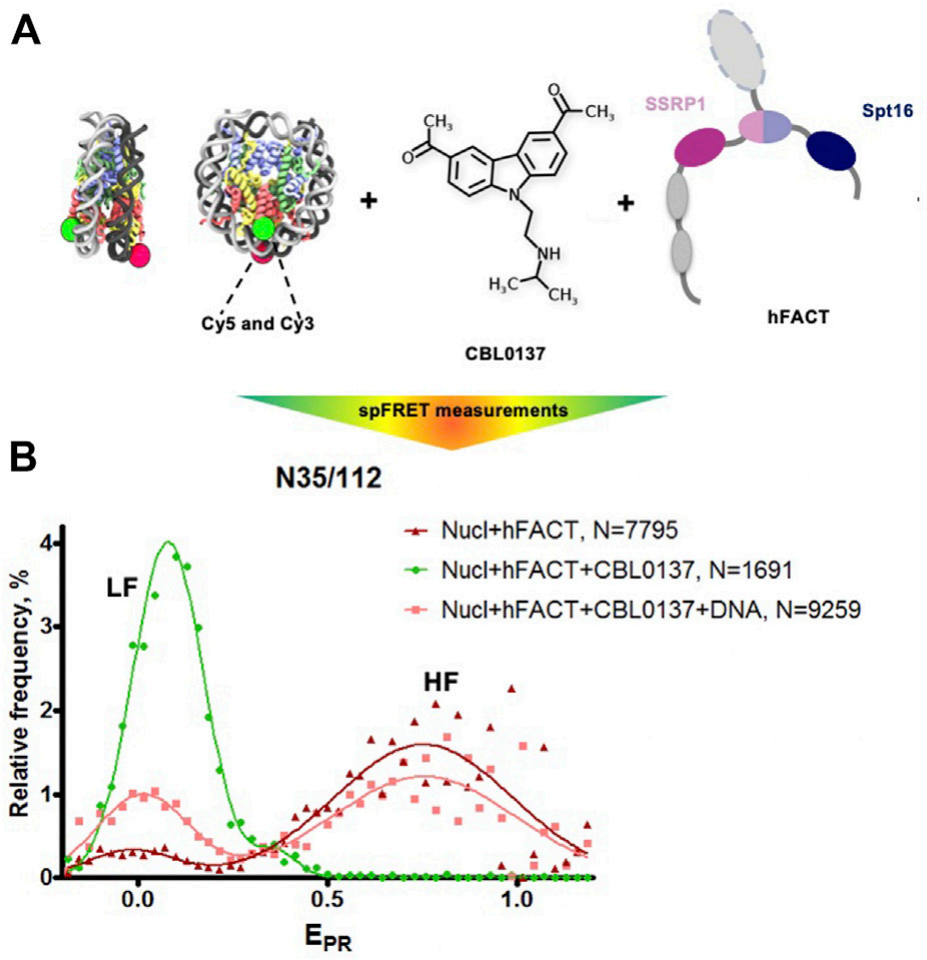
FACT and curaxin CBL0137 work synergistically and induce a large-scale nucleosome unfolding. **(A)** Experimental approach for analysis of nucleosome unfolding using spFRET. Mononucleosomes contained a single pair of Cy3 and Cy5 dyes in the nucleosomal DNA (shown as green and red circles, respectively), **(B)** Nucleosome unfolding by FACT/CBL0137 under conditions used for electron microscopy: analysis by spFRET microscopy. Frequency distributions of FRET efficiencies (E_PR_) in the presence of FACT and/or CBL0137 and competitor DNA. Gaussian peaks having lower and higher FRET efficiencies are indicated (LF and HF, respectively).

The interaction of FACT with nucleosomes was studied using mononucleosomes assembled on the 603 Widom nucleosome positioning sequence (Thastrom et al., 1999). Nucleosomal DNA contained a single pair of Cy3 and Cy5 fluorophores in positions 35 and 112 bp from the nucleosomal entry/exit boundary, allowing fluorescence resonance energy transfer (FRET) between the fluorophores and detection of the conformation changes in nucleosomal DNA upon interaction with FACT and curaxins (Valieva et al., 2016; Chang et al., 2018). Single particle FRET (spFRET) from the nucleosomes was measured in the absence and presence of curaxin CBL0137, FACT and competitor DNA (Figure 5A).

As expected, no changes in nucleosome structure were detected in the presence of FACT alone and only minor increase of the height of the low-FRET peak and corresponding decrease of the high-FRET peak were detected in the presence of CBL0137 only (Supplementary Figure S3). In contrast, FACT and CBL0137 added to the nucleosomes together induced a profound transition from high to low FRET, reflecting a dramatic uncoiling of nucleosomal DNA (Figure 5B and Supplementary Figure S3) (Valieva et al., 2016; Chang et al., 2018). These changes in the nucleosomal DNA were largely reversed by subsequent addition of an excess of competitor DNA that removes FACT from the complex. Thus, FACT induces a large-scale, reversible nucleosome unfolding in the presence of curaxin (Chang et al., 2018; Chang et al., 2019), consistent with our previous results obtained using electrophoresis mobility shift assay (EMSA) (Chang et al., 2018).

To directly visualize the process of nucleosome unfolding by FACT in the presence of CBL0137, the complexes of FACT with nucleosomes were formed in the presence of CBL0137, characterized by spFRET microscopy immediately before EM (Figure 5B), applied to the EM grid, negatively stained and studied using TEM. Single particle images were collected using a neural network in EMAN2.3 (Tang et al., 2007) and subjected to 2D-classification in RELION2.1 (Supplementary Figures S4, S5).

In the sample that contains FACT and nucleosomes in the absence of curaxin the following class-average complexes were detected after 2D classification (Supplementary Figure S4): 1) nucleosomes (an excess of nucleosomes was added to minimize the presence of nucleosome-free FACT), 2) nucleosome-free FACT present in the open, compact and closed conformations, and 3) folded FACT-nucleosome complexes.

**FIGURE 6:**
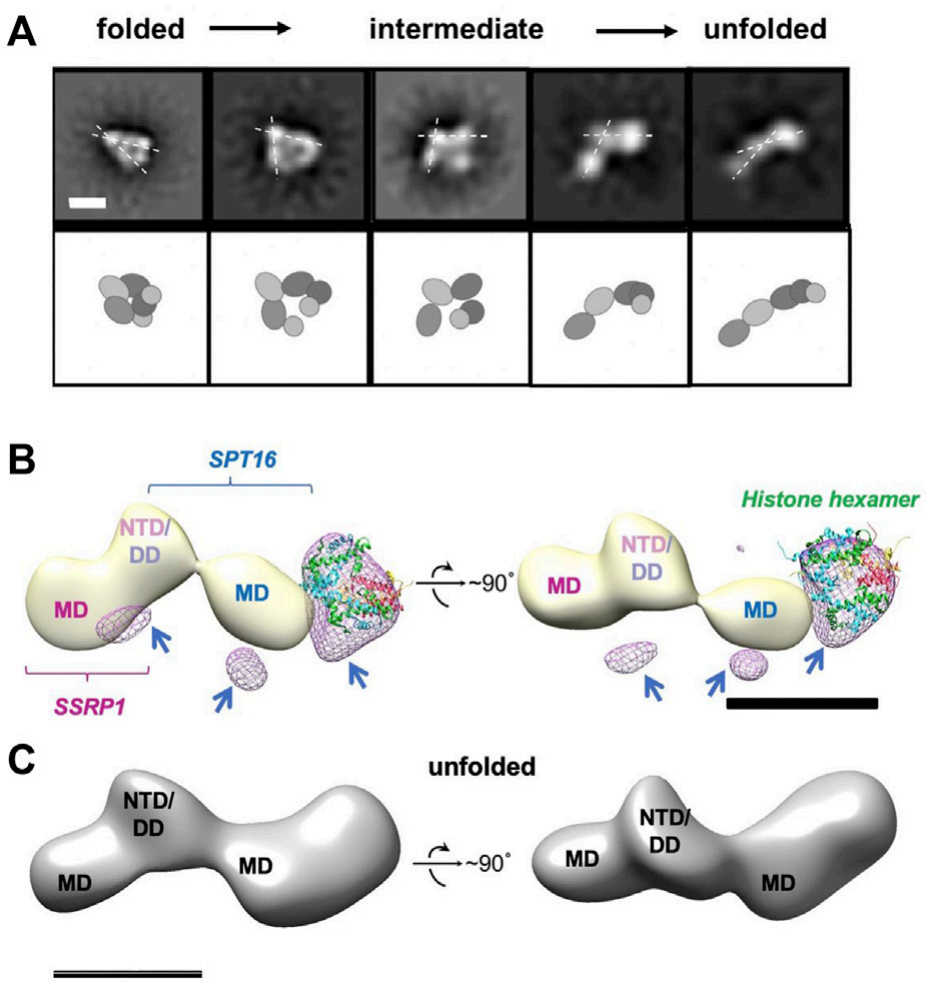
Unfolding of FACT-nucleosome complex in presence of curaxin CBL0137. **(A)** Step-by-step complex unwrapping by FACT in the presence of CBL0137. Top: Characteristic 2D class-averages of FACT-nucleosome complexes (in the presence of CBL0137). Dashed lines indicate the angle between SSRP1 MD-NTD/SPT16 DD-MD domains, which increases during nucleosome unfolding. Bar—10 nm. Bottom: Schematic of the proposed domain rearrangements as shown in the upper panel, **(B)** Difference map between unfolded FACT-nucleosome complex and FACT in the open state (light yellow); the resulting differential density is shown in magenta mesh. Histone hexamers (H2A/H2B dimer and H3/H4 tetramer derived from 6UPK PDB) were fitted into the differential density with correlation coefficient of 0.88; arrows are pointing to the additional differential densities, which can be attributed to the DNA bound to the complex; **(C)** Model of the unfolded FACT-nucleosome complex. Hypothetical position of DNA (purple) is based on the assumption that both SPT16-DD/MD and SSRP1-DD/MD domains maintain their interactions with DNA previously observed in the compact complex (Liu et al., 2020).

Adding curaxins to the FACT-nucleosome complex resulted in formation of several novel conformations of the complex (Figure 6A and Supplementary Figure S5), which are likely to represent intermediates formed during stepwise nucleosome unfolding. The set of panels on Supplementary Figure S5 show structures smaller than 10 nm, i.e. less than either free FACT or nucleosome diameters. These could be particle contaminations present in the sample. Multiple intermediates between the initial folded and fully unfolded complexes were identified (Figure 6A and Supplementary Figure S6); the length of the intermediates spanned the range from 17.3 ± 2.3 to 21.6 ± 2.5 nm.

Importantly, the 2D projections of the folded FACT-nucleosome complexes closely resembled those obtained by Liu et al. (2020) (Supplemenatry Figure S8A), although entirely different strategies for assembly of the complexes were used. This observation allowed reconstruction of the 3D map of the compact FACT-nucleosome complexes using RELION 3.0; the 3D model was built using 19,074 particles with a final resolution of 22 Å (Supplementary Figure S8B). The previously determined atomic structure of the folded FACT-nucleosome complex (Liu et al., 2020) was fitted in the observed 3D electron density with the correlation coefficient of 0.92, indicating similar organization of the complexes.

The images of the unfolded complexes were extracted from the dataset and used for 3D reconstruction of the unfolded complex in RELION2.1 (Figure 6C). The reconstruction has a clear four-density map, with three densities similar in size to corresponding densities of FACT in the open conformation (compare Figures 2B, 6A), and the additional fourth domain (Figure 6B). The fourth domain can accommodate the H3/H4 tetramer and possibly one H2A/H2B dimer (linear dimensions are ∼10 × 5 nm).

As we have shown previously for yFACT unfolding the nucleosome together with yeast Nhp6 protein (Sivkina, 2022), short ∼35-bp regions of nucleosomal DNA were missing on both sides of nucleosome-FACT density after nucleosome unfolding. Here we also did not detect nucleosomal DNA in our 3D reconstructions, likely because these flexible regions were averaged during image processing. To ensure that DNA is retained in the nucleosomes unfolded in the presence of FACT and curaxin, we analyzed the raw images used to obtain 2D projections of partially and completely unfolded complexes. On the raw images the extra densities extending from the sides of the main FACT-histones density are visible; as expected, they demonstrate a higher flexibility than the densities that are better resolved in corresponding 2D projections (Supplementary Figure S9).

The second H2A/H2B dimer could remain in contact with SSRP1-MD domain, stabilized by SSRP1-CID region and flexibly linked to nucleosomal DNA (Farnung et al., 2021)*,* preventing it from being resolved in the open complex. Other FACT domains (SPT16 CTD, SSRP1 IDD&HMG&CID) are also unlikely to be ordered sufficiently to be resolved (Liu et al., 2020).

In summary, binding of FACT to the nucleosome in the presence of curaxin CBL0137 induced a dramatic unfolding of nucleosomal DNA that was accompanied by formation of a multi-density complex containing core histones and both subunits of FACT. The complex is a mixture of intermediates that contain nucleosomes unfolded to different degrees. The most folded complex is structurally similar to the FACT-nucleosome complex characterized previously (Liu et al., 2020); the similarity allowed assignment of electron densities in the folded complex to various FACT domains and core histones. Subsequent analysis of the unfolded intermediates suggests a pathway of progressive curaxin-dependent nucleosome unfolding by FACT.

### 2.4 Mechanism of FACT/curaxin-dependent nucleosome unfolding

**FIGURE 7:**
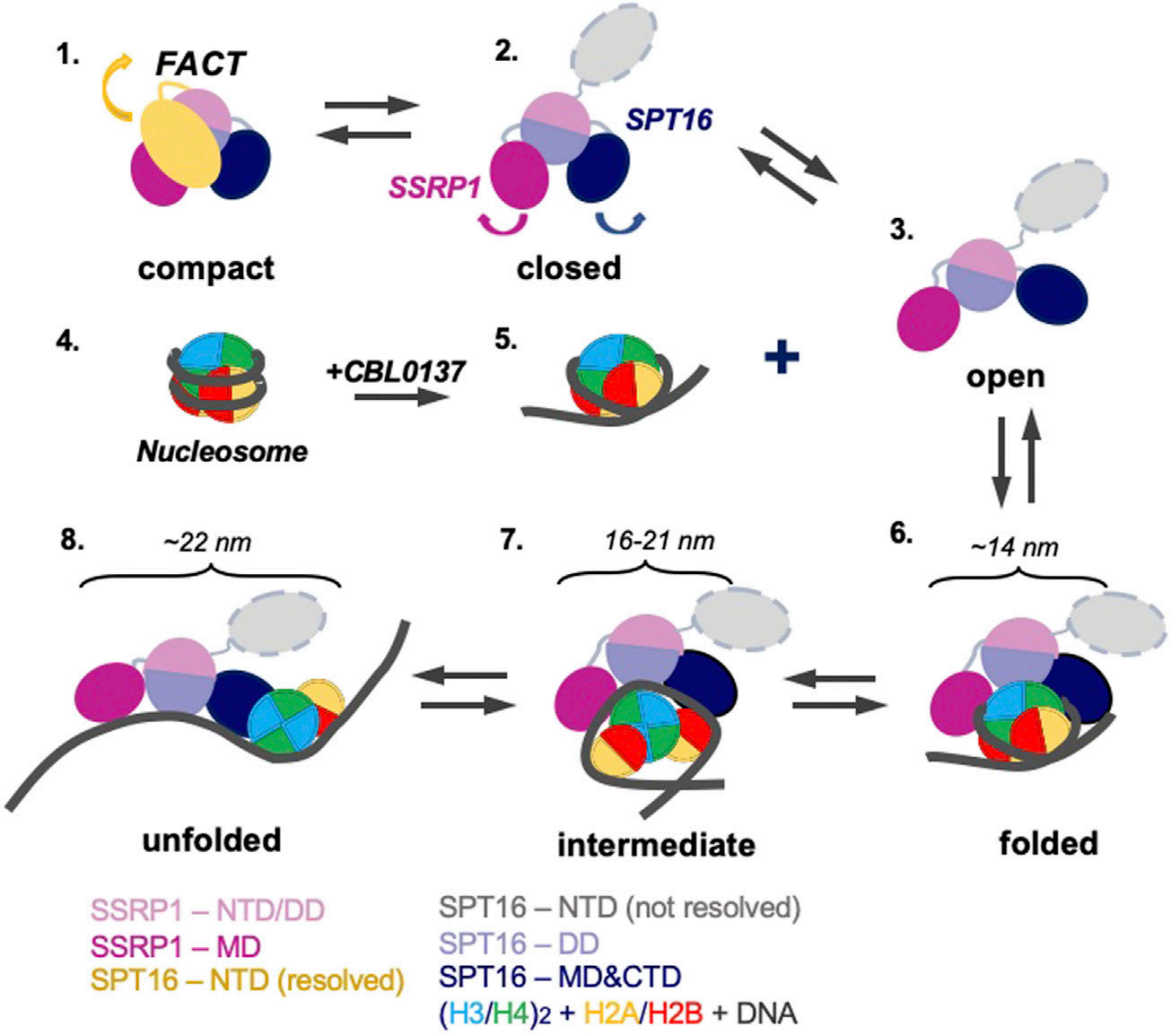
Model of nucleosome unfolding by FACT in the presence of curaxin CBL0137. FACT is a mixture of compact, closed and open states (intermediates 1, 2, and 3). As nucleosomal DNA is partially uncoiled from the histone octamer in the presence of CBL0137 (intermediates 4 and 5), FACT-binding sites on the surface of H2A/H2B dimers become available for interaction with FACT, and the folded complex is formed (intermediate 6). As a result, FACT induces further nucleosome unfolding (intermediates 7 and 8). The linear dimensions of the intermediates 6 and 7 are indicated. The color code is shown at the bottom.

The data described above suggest the following scenario for nucleosome unfolding by FACT in presence of curaxin (Figure 7). Nucleosome-free FACT a mixture of compact, closed and open states (Figure 1B). In the compact conformation of the complex, the C-terminal DNA-binding regions of both subunits of FACT could interact with other domains of FACT (Sivkina et al., 2022) and the DNA-binding domains on the SPT16 and SSRP1 subunits are likely hidden and not available for interaction with a nucleosome.

The comparison of 3D maps of FACT (Figures 2B,C) suggests that opening of the complex likely occurs through a concerted movement of the four domains. First, SPT16-NTD moves away from the complex and thus probably “unlocks” the mobilities of the other domains (Figure 7, intermediates 1 and 2); then other domains of FACT move away from each other, forming the open complex (Figure 7, intermediate 3). In the open conformation of FACT the DNA-binding sites on the dimerization and middle domains of SPT16 and SSRP1 subunits, respectively (Liu et al., 2020), as well as the C-terminal DNA-binding regions of both subunits of FACT (Sivkina et al., 2022) become available for interaction with nucleosomes.

However, FACT interacts weakly with intact nucleosomes; the DNA at the entry/exit sites has to be partially displaced from the histone octamer to expose binding sites for FACT, which was accomplished by removing this DNA in the cryo-EM map (Liu et al., 2020) or by adding high levels of the HMBG factor Nhp6 (Sivkina et al., 2022)*.* Our results show that curaxin CBL0137 provides this activity as well (Figure 7, intermediates 4 and 5). This DNA intercalator (Safina et al., 2017) binds to and induces partial displacement of the nucleosomal DNA from the octamer (Figure 2B and (Chang et al., 2018)), exposing the FACT-binding surfaces on H2A-H2B dimers. FACT binds to the destabilized nucleosome and the folded complex is formed (Figure 7, intermediate 6); the complex is structurally similar with the FACT-nucleosome complex described previously (Liu et al., 2020).

The initial binding of FACT to the nucleosome triggers a progressive sequence of events leading to formation of the intermediate and unfolded complexes (Figure 5, intermediates 7 and 8) containing nearly completely uncoiled nucleosomal DNA. Since FACT-dependent nucleosome unfolding is an ATP-independent process (Chang et al., 2018; Sivkina et al., 2022), it most likely occurs through a set of intermediates (Figure 6A) having similar free energies that are reversibly interconverted. For each pair of the intermediates the equilibrium can be easily shifted in either direction by engaging additional protein-protein and/or DNA-protein interactions (Sivkina et al., 2022), or through partial uncoiling of nucleosomal DNA from the octamer by curaxins.

The unfolded complex is stabilized by multiple interactions of different FACT domains with both nucleosomal DNA and core histones (Figure 6D). Since each of these interactions is relatively weak, nucleosome unfolding is a partially reversible process: thus, intact nucleosomes can be largely recovered in the presence of competitor DNA (Figure 5B) that presumably binds and outcompetes the curaxin from the nucleosomal DNA. Upon the removal of the curaxin nucleosomal DNA re-binds to core histones and FACT dissociates from the complexes.

## 3 Discussion

Our structural analysis of the process of curaxin-dependent nucleosome unfolding by FACT using spFRET and TEM revealed that in the absence of nucleosomes FACT is a flexible complex that exists in compact, closed and open conformations present at the ratio of 43:22:35, respectively (Figure 1). Four or three distinct densities are visible in the compact and closed/open conformations, respectively; molecular modeling allowed assignment of these electron densities to FACT domains (Figure 2). The arrangement of the densities in the complexes suggests that SPT16-NTD domain “locks” the other resolved domains of FACT in the compact conformation (Figure 2D). While FACT alone binds weakly to a nucleosome, multiple structurally different FACT-nucleosome complexes (folded, intermediate and unfolded) are formed in the presence of curaxin CBL0137 (Figure 5). Electron densities in the folded complex were assigned to several FACT domains and core histones (Supplementary Figure S8). Molecular modeling suggests that the unfolded complex contains nearly linear DNA, core histones and both subunits of FACT (Figures 6C,D). Subsequent analysis of the unfolded intermediates suggests a pathway of progressive curaxin-dependent nucleosome unfolding by FACT (Figure 7).

Similar distributions between the open and closed conformations for yeast (Sivkina et al., 2022) and human FACT in the absence of other factors (∼35:65, Supplementary Figure S7) highlight the overall structural similarity of these factors. At the same time, the pathways of nucleosome unfolding by yeast FACT in the presence of the DNA-binding protein Nhp6 (Sivkina et al., 2022) and by human FACT in the presence of curaxin are different. In both cases, complete nucleosome unfolding requires the presence of all participating factors. However, the curaxin interacts with DNA and therefore the observed increase in the presence of the open FACT complexes after the nucleosome unfolding from 35% to 44% (Supplementary Figure S7A) likely occurs only due to nucleosome destabilization by curaxin (Supplementary Figure S3). In contrast, Nhp6 protein interacts both with FACT and with nucleosomes (Sivkina et al., 2022), and therefore induces an increase in the fraction of the open forms of FACT both in the absence and in the presence of nucleosomes (36–51% and 55%, respectively, Supplementary Figure S7B). Accordingly, the overall efficiencies of nucleosome unfolding by FACT with curaxin and with Nhp6 protein are different (44% *vs.* 55%, respectively).

**FIGURE 8:**
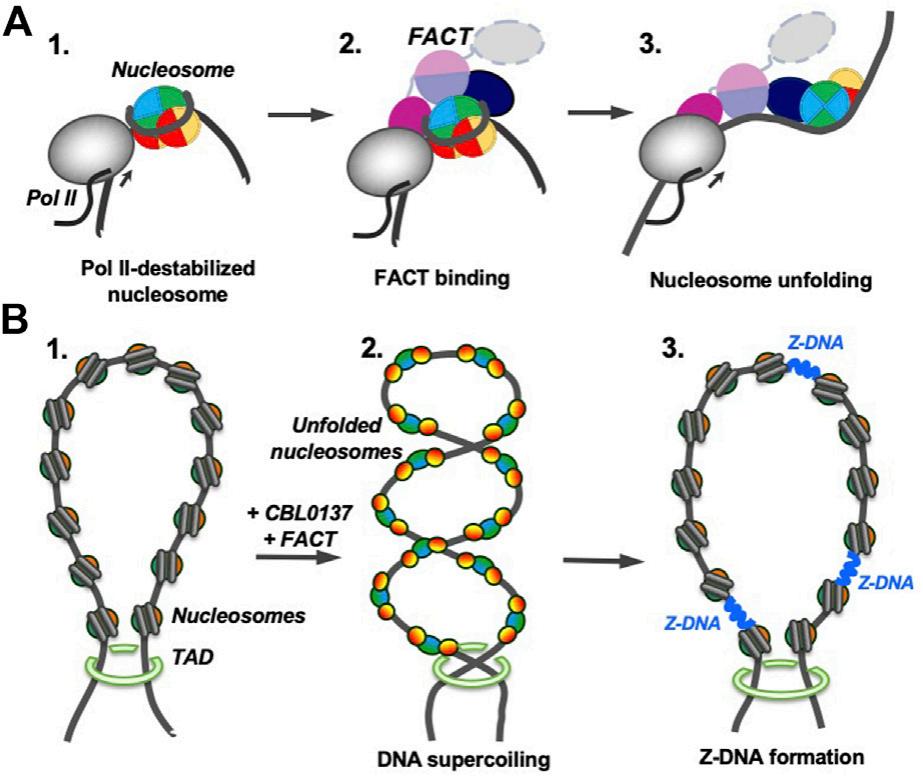
Possible functions of FACT-induced nucleosome unfolding. The color code as in Figure 7. **(A)** FACT-dependent nucleosome unfolding during transcription by Pol II. Transcribing RNA polymerase II (Pol II) partially uncoils nucleosomal DNA from the histone octamer and opens FACT-interacting surfaces of H2A/H2B dimers (intermediate 1) (Kulaeva et al., 2009). FACT binds to the destabilized nucleosomes (intermediate 2) and induces nucleosome unfolding (intermediate 3) that could facilitate further transcription and nucleosome survival during this process. **(B)** Curaxin-dependent Z-DNA formation. As curaxins and FACT interact with nucleosomes in a chromatin region, that is, closed by topologically associating domains (TADs, structure 1), bulk nucleosomes are unfolded, releasing unconstrained DNA supercoiling (intermediate 2). DNA supercoiling, in turn, induces formation of Z-DNA (intermediate 3) that could serve as a trigger of necroptosis in cancer cells (Safina et al., 2017; Singh et al., 2022).

Comparison of the curaxin- and Nhp6-dependent pathways also suggests that partial uncoiling of nucleosomal DNA from the histone octamer is a necessary pre-requisite for nucleosome unfolding by FACT. Partial DNA uncoiling that must occur during transcription and replication exposes FACT-binding sites on the octamer and provides a target for FACT binding. Indeed, FACT is associated with transcribed genes and the replication fork (Belotserkovskaya et al., 2003; Hsieh et al., 2013; Kulaeva et al., 2013; Safaric et al., 2022); since destabilized nucleosomes have exposed binding sites for FACT, nucleosome unfolding likely occurs during these processes (Figure 8A). It has been proposed that nucleosome unfolding could facilitate nucleosome survival during transcription (van Holde et al., 1992); although there is no direct evidence for unfolding of these nucleosomes, FACT facilitates nucleosome survival during transcription *in vitro* (Hsieh et al., 2013). Therefore, FACT could possibly induce nucleosome unfolding and this could support nucleosome survival (Figure 8A).

At the same time, FACT is known for its ability to both destabilize and assemble nucleosomes. Formation of energetically similar intermediates during FACT-dependent nucleosome unfolding (Figure 7, intermediates 6–8) can explain this apparent contradiction in properties. Indeed, the equilibrium between the intermediates can be easily shifted in either direction in processes involving relatively low energy cost, such as formation of several additional DNA-protein interactions by Nhp6 protein (Sivkina et al., 2022) or curaxin intercalation into nucleosomal DNA (this work). These factors induce FACT-dependent nucleosome unfolding; an example of the process developing in the opposite direction is reversal of FACT-dependent nucleosome unfolding in the presence of competitor DNA (Figure 5B). None of these factors (Nhp6, curaxin or competitor DNA) strongly affects intact nucleosomes; all of them work highly synergistically with FACT. In all cases FACT creates an intermediate that serves as a “decision point” allowing either nucleosome unfolding or recovery after an additional interaction of the factors with DNA or a protein.

Curaxins reduce the growth of cancer cells by inducing the trapping of FACT within bulk chromatin (c-trapping) (Gasparian et al., 2011; Chang et al., 2018). The trapping most likely occurs because human FACT binds weakly to intact nucleosomes (Supplementary Figure S6) but has higher affinity for partially unwound nucleosomes. Curaxin-induced binding of FACT to bulk nucleosomes (Supplementary Figure S6) most likely explains the observed re-distribution of FACT from transcribed genes to bulk chromatin (Chang et al., 2018). Importantly, the dramatic curaxin- and FACT-induced nucleosome unfolding (Figure 5) likely changes the global arrangement of topologically closed chromatin loops, introducing unconstrained negative DNA supercoiling (Chang et al., 2019). The changes in chromatin topology likely contribute to the observed genome-wide formation of Z-DNA in cancer cells in the presence of curaxins (Safina et al., 2017; Zhang et al, 2022) (Figure 8B).

A limitation of our electron microscopy approach is the use of negative stain that was used because the analyzed molecules are highly flexible (Sivkina et al., 2022); therefore the resolution is limited by the contraster grain size. However, since the critical 3D maps obtained in our studies are very similar with the 3D maps obtained using more preservative electron cryo-microscopy (Supplementary Figure S8A) the molecular details of the mechanism can be derived with confidence.

FACT complex and ATP-dependent chromatin remodeling complexes participate in the nuclear processes involving the nucleosome eviction (Ray-Gallet & Almouzni, 2022; Safaric et al., 2022). Therefore, curaxins cause the destabilization of nucleosomal structure and possibly could stimulate nucleosome unfolding or sliding by other remodelers as well as by FACT.

In summary, FACT is a remarkably flexible protein complex; its flexibility is an important factor during FACT-dependent nucleosome unfolding where “decision point” intermediates are formed. The equilibrium between the intermediates can be shifted in either direction with relatively low energy cost. Similar intermediates are likely formed during transcription and replication in the cell nuclei; their formation allows FACT to either destabilize or assemble nucleosomes, depending on the presence of additional factors. In particular, curaxins induce reversible FACT-dependent nucleosome unfolding, likely through intercalation into nucleosomal DNA and destabilization of nucleosomes, leading to FACT trapping in bulk chromatin in cancer cells.

## 4 Materials and methods

### 4.1 Experimental design

The objectives of the study were to determine the molecular mechanism of curaxin-dependent nucleosome unfolding by FACT. The experimental design includes use of highly purified components (core nucleosomes, curaxin CBL0137 and FACT) and experimental approaches allowing detailed analysis of highly flexible complexes (electron microscopy and spFRET).

### 4.2 Protein purification

-H1 chicken erythrocyte chromatin and chicken nucleosome cores were purified as described (Gaykalova et al., 2009). Recombinant FACT subunits were co-expressed in insect Sf9 cells as described (Belotserkovskaya et al., 2003) and FACT was purified as described (Hsieh et al., 2013). Recombinant SPT16/SSRP1ΔNTD complex was expressed as a dimer in *E. coli* and purified as described (Valieva et al., 2016).

### 4.3 Nucleosome assembly and purification

A plasmid containing the modified 603–42 nucleosome positioning sequence (Kulaeva et al., 2009) was used to obtain the nucleosomal DNA template by PCR with the following fluorescently labeled primers:

#### 4.3.1 Forward primer

5′–CCC​GGT​TCG​CGC​TCC​CTC​CTT​CCG​TGT​GTT​GTC​GT*CTC​T-3′

(where T*—is a nucleotide labeled with Cy5);

#### 4.3.2 Reverse primer

5′–ACC​CCA​GGG​ACT​TGA​AGT​AAT​AAG​GAC​GGA​GGG​CCT#CTT​TCA​ACA​TCG​AT-3′

(where T#—is a nucleotide labeled with Cy3).

The 147-bp N35/112 DNA fragments were purified using Evrogen Cleanup Standart kit (Evrogen, Russia).

Nucleosomes were assembled by octamer transfer from -H1 chromatin to DNA templates after dialysis from 1M NaCl to 0.01M NaCl as described (Kireeva et al., 2002; Gaykalova et al., 2009). For gel shift analysis of FACT binding and spFRET analysis nucleosomes were purified by PAGE under non-denaturing conditions as described (Studitsky, 1999; Valieva et al., 2016).

For spFRET experiments N35/112 nucleosomes were gel purified and analysed at a concentration of 0.5–1 nM after incubation in the presence of FACT (0.1 μM) and/or CBL0137 (2 μM) in the buffer containing 20 mM Tris-HCl pH7.9, 150 mM KCl for 5 min at 25°C.

### 4.4 Single particle FRET experiments

SpFRET measurements in solution and analysis were performed as described (Kudryashova et al., 2015; Valieva et al., 2016). The proximity ratio E_PR_ was calculated as.

$$E_{\mathrm{PR}}=\left(I_{a}\;–0.19\times I_{d}\right)\;/\left(I_{a}+0.81\times I_{d}\right)$$ (1)

where I_a_ and I_d_ are Cy5 and Cy3 fluorescence intensities corrected for background. Factors 0.19 and 0.81 were introduced to correct for the contribution of Cy3 fluorescence in the Cy5 detection channel (spectral cross-talk) (Kudryashova et al., 2015).

Proximity ratios E_PR_ were calculated using 800–8000 signals from single nucleosomes for each measured sample and plotted as a relative frequency distribution. Each plot was fitted with a sum of two Gaussians to describe two conformational states of nucleosomes. The fractions of nucleosomes in different states were estimated as the areas under the corresponding Gaussian peaks normalized to the total area of a plot by using LabSpec program. Reproducibility of the results was verified in at least three independent experiments.

### 4.5 Preparation of samples for electron microscopy

SPT16/SSRP1 and SPT16/SSRP1ΔNTD complexes were prepared in previously characterized buffer containing 17 mM HEPES pH 7.6, 2 mM Tris-HCl pH 7.5, 0.8 mM Na3EDTA, 0.11 mM 2-mercaptoethanol, 11 mM NaCl, and 1.1% glycerin, 12% sucrose (Hsieh et al., 2013; Valieva et al., 2016; Sivkina et al., 2022) at concentration of 0.05 μM.

Complexes of FACT with the nucleosome were formed in the presence of 0.1 μM FACT, 0.1 μM core chicken nucleosomes, 2 μM CBL0137, and 0.5 nM fluorescently labeled core nucleosomes N35/112 on ice for 42 h. Since complexes of human FACT with nucleosomes are not stable in a native gel but stable in solution (Chang et al., 2018), the complexes were analyzed using spFRET and TEM. Part of the sample was used to evaluate reversibility of nucleosome reorganization by adding salmon sperm DNA to final concentration 0.65 μg/μl for 0.5 h on ice, followed by spFRET-microscopy. The remaining sample was used for TEM.

### 4.6 Transmission electron microscopy and image analysis

Protein samples and complexes were applied to the carbon-coated glow-discharged in Emitech K100X device (Emitech Ltd., United Kingdom) copper grid (Ted Pella, United States) immediately after preparation, subjected to glow-discharge using Emitech K100X device (Emitech Ltd., United Kingdom), stained for 30 s with 1% uranyl acetate, and air dried. Grids were studied in JEOL 2100 TEM (JEOL) microscope operated at 200 kV at low-dose conditions. Micrographs were captured by the Gatan Ultrascan camera with magnification ×25,000, no tilt, with 4.1 Å pixel size using SerialEM software (de la Cruz et al., 2019; Schorb et al., 2019)*.*

Single particle images of FACT, complexes of FACT with the nucleosome and complexes of FACT with the nucleosome formed in the presence of CBL0137 were collected from the micrographs using a neural network provided by EMAN2.3 software. Single particles coordinates collected by the neural network were imported in RELION2.1 software; all further 2D-processing, analysis and CTF-correction were performed using RELION2.1 software. Extracted particles were used for iterative 2D-classification followed by the elimination of bad classes. Consolidated information for the analyzed data (micrographs, particles, numbers of classes) is presented in Supplementary Table S2. Linear dimensions of the 2D-classes were measured with ImageJ (Abràmoff etal, 2004). Initial 3D reconstitution was performed in EMAN2.3 (Tang et al., 2007) using selected classes representing different views of the particles. Initial 3D-models were imported to RELION 2.1 and used as a reference for 3D classification, followed by auto-refinement, masking, post-processing and determination of final resolution in this program. All 3D reconstructions were visualized and analyzed in UCSF Chimera (Pettersen et al., 2004). Analysis of the FACT-ΔNTD mutant was conducted using the same steps, except single particle images were collected using autopicking utility in RELION3.0 software.

### 4.7 Structural analysis of the interacting domains in the compact FACT conformation

The model of the three-domain FACT complex was constructed based on the crystal structure of FACT-nucleosome complex 2 [pdb id 6upl (Liu et al., 2020)]. The model of unfolded FACT-nucleosome complex was built using the atomic structure of the folded complex [pdb id 6upl (Liu et al., 2020)], followed by rigid fitting of the domains with DNA in the map of electron density of the complex using USCF Chimera software. The structure of *Homo sapiens* NTD-Spt16 domain was downloaded from rscb.org [pdb id 5e5b (Marciano and Huang, 2016)]. The hydrophobic organization of interacting subunits in compact four-domains structure FACT was analyzed using Platinum web service (Pyrkov et al., 2009) and UCSF Chimera (Pettersen et al., 2004). The data on hydrophobic organization of interacting subunits were used to determine the initial orientations of the domains for flexible molecular docking *via* HADDOCK 2.4 server (Dominguez et al., 2003). Based on the HADDOCK score and RMSD (root mean square deviation) all conformations were divided into clusters and were visually analyzed in UCSF Chimera.

### 4.8 Molecular dynamics

Molecular dynamics (MD) simulation was performed as described (43). Starting models of the complex was constructed using HADDOCK (41). The Gromacs package (44) and OPLS forcefield (45) were used for the simulations. After the relaxation of the systems, the last 10 ns of trajectory were used for analysis.

The contacts between subunits of FACT were evaluated using the Protein Interactions Calculator (PIC) server (Tina et al., 2007).

### 4.9 Statistical analysis

In spFRET measurements, the E_PR_ profiles and contents of nucleosome subpopulations were averaged (mean ± SEM) over three independent experiments. The sample sizes varied from 1600 to 8800 particles per each independent experiment.

In electron microscopy experiments, fractions of open and closed complexes were calculated as the average of three experiments.

## Data Availability

The datasets presented in this study can be found in online repositories. The names of the repository/repositories and accession number(s) can be found in the article/Supplementary Material.
