# Supplementary material for: Mechanism of curaxin-dependent nucleosome unfolding by FACT
Source: Front Mol Biosci. 2022 Nov 22;9:1048117. doi: 10.3389/fmolb.2022.1048117 (PMC9723464; doi:10.3389/fmolb.2022.1048117)
Supplement: Supplementary file 1 [file DataSheet1.pdf]

Supplementary Materials for

Mechanism of Curaxin-dependent Nucleosome Unfolding by FACT

O. I. Volokh, A.L. Sivkina, A.V. Moiseenko, A.V. Popinako, M. G. Karlova, M.E. Valieva, E. Y. Kotova, M. P. Kirpichnikov, T. Formosa, V. M. Studitsky*, O.S. Sokolova*

*Corresponding authors. *sokolova@mail.bio.msu.ru*; *vasily.studitsky@fccc.edu*

This PDF file includes:

Figs. S1 to S9
Tables S1 to S2

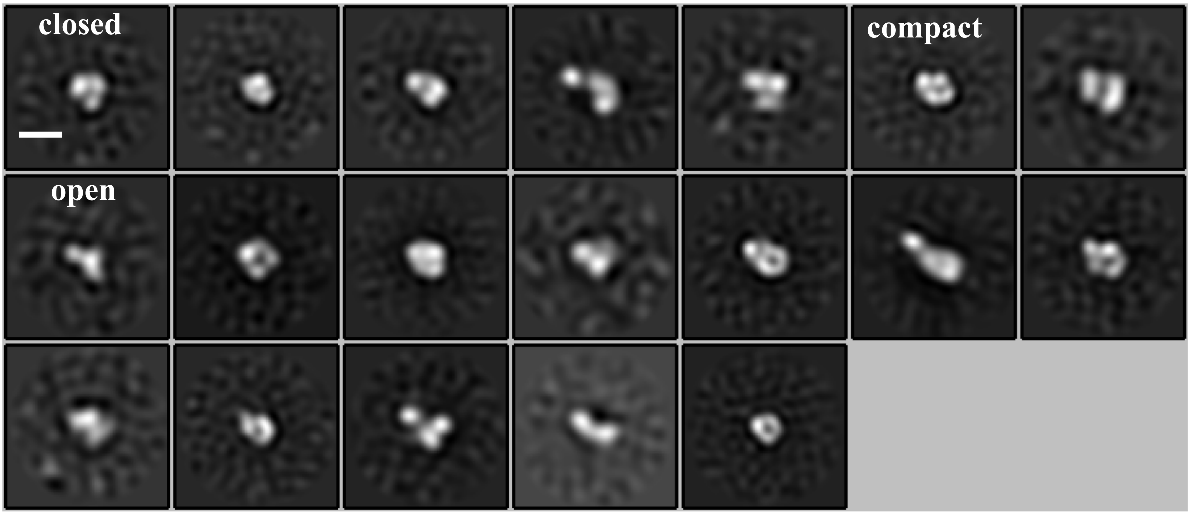

Fig. S1. 2D class-averages of FACT complexes.
Three distinct conformations (compact, closed and open) were identified. Bar – 10 nm.

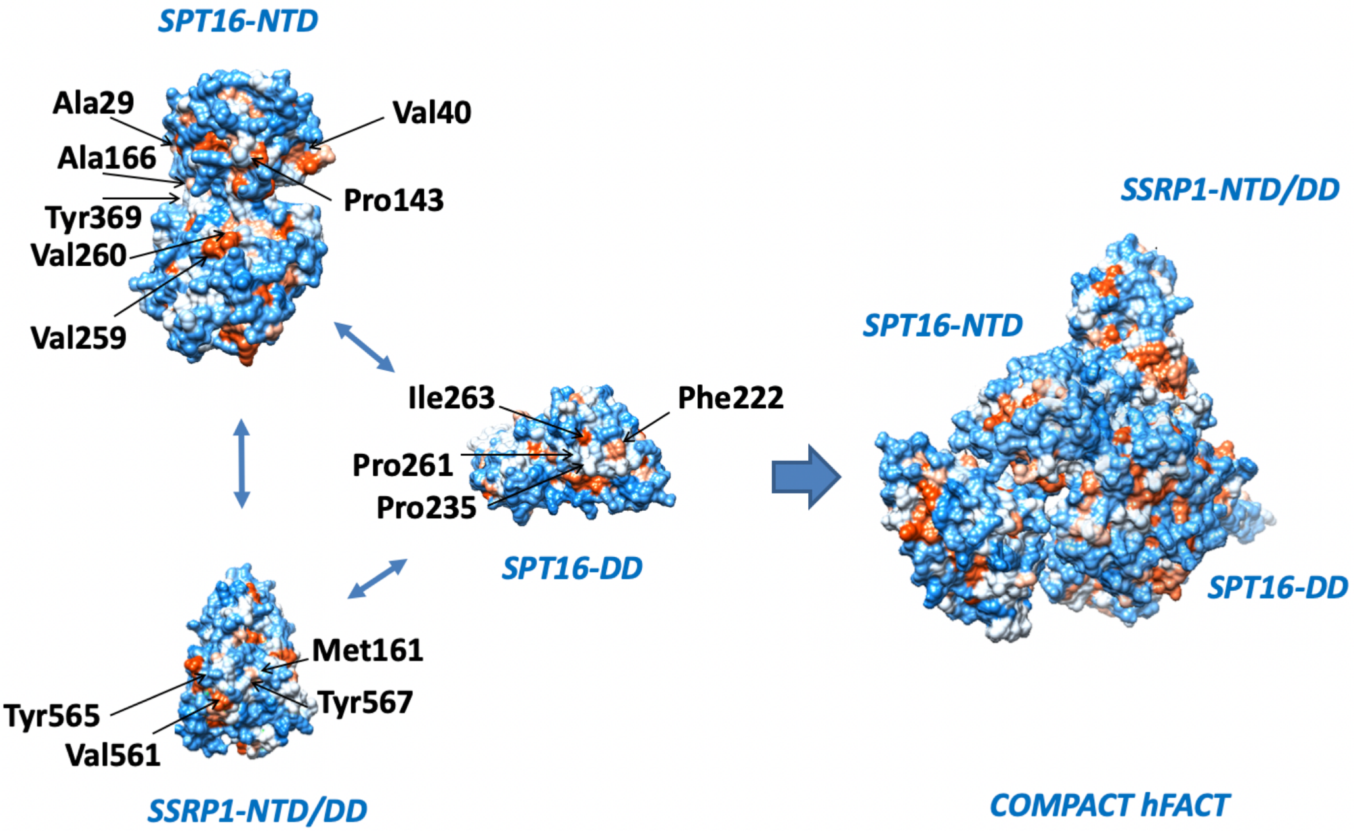

Fig. S2. Surfaces participating in interactions between domains of human FACT subunits.
The interacting surfaces of different FACT domains are shown on the left and the entire compact FACT structure in the compact conformation – on the right. Hydrophobic and hydrophilic regions are shown in orange and blue, respectively. The key amino acids involved in the interactions are indicated.

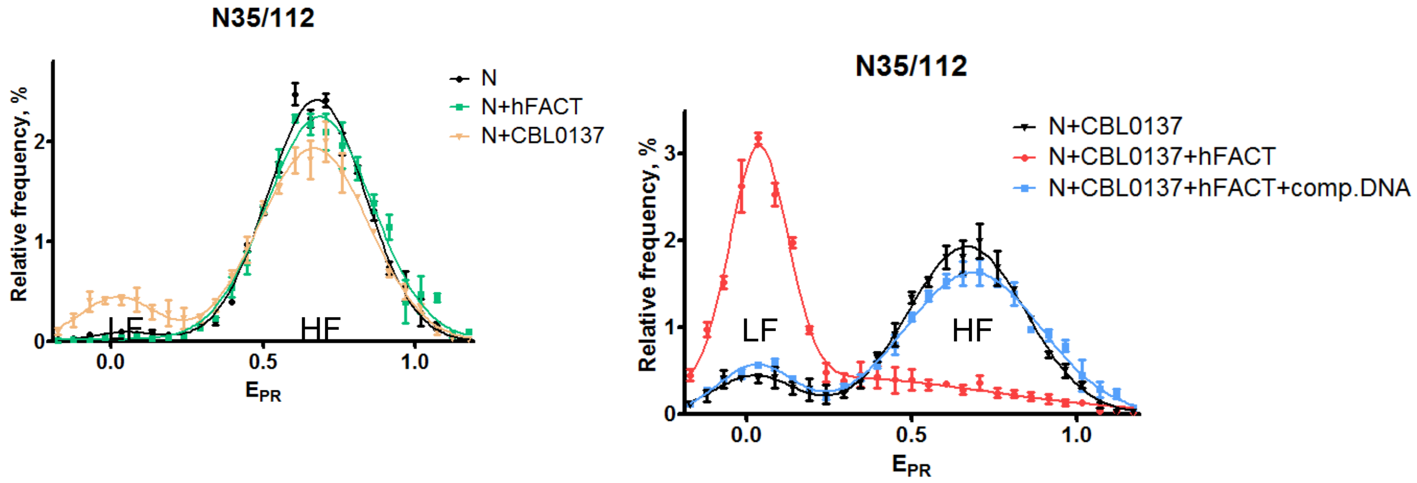

Fig. S3. FACT and curaxin CBL0137 work synergistically and induce a large-scale, reversible nucleosome reorganization.

Typical frequency distributions of FRET efficiencies (EPR) in the presence or absence of curaxin CBL0137, FACT and/or competitor DNA. Analysis by spFRET microscopy. The mean values of EPR peaks and the standard errors were the following: (N) – 0.057±0.063, 0.676±0.004; (N+FACT) – 0.073±0.093, 0.688±0.014; (N+CBL0137) – 0.027±0.048, 0.669±0.012; (N+CBL0137+FACT) – 0.036±0.005, 0.525±0.09; (N+CBL0137+FACT+competitor DNA) – 0.026±0.005, 0.687±0.008. Low-FRET and high-FRET peaks are indicated as HF and LF, respectively.

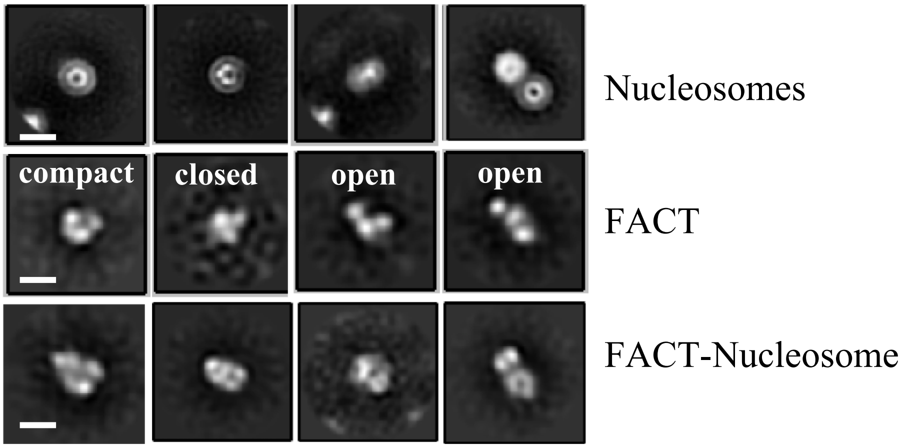

Fig. S4. Representative 2D class-averages of complexes formed in the presence of FACT and nucleosomes without curaxin CBL0137.

Middle raw - three distinct conformations (compact, closed and open) of FACT. Bottom raw – different views of the folded FACT-nucleosome complex. Bar – 10 nm.

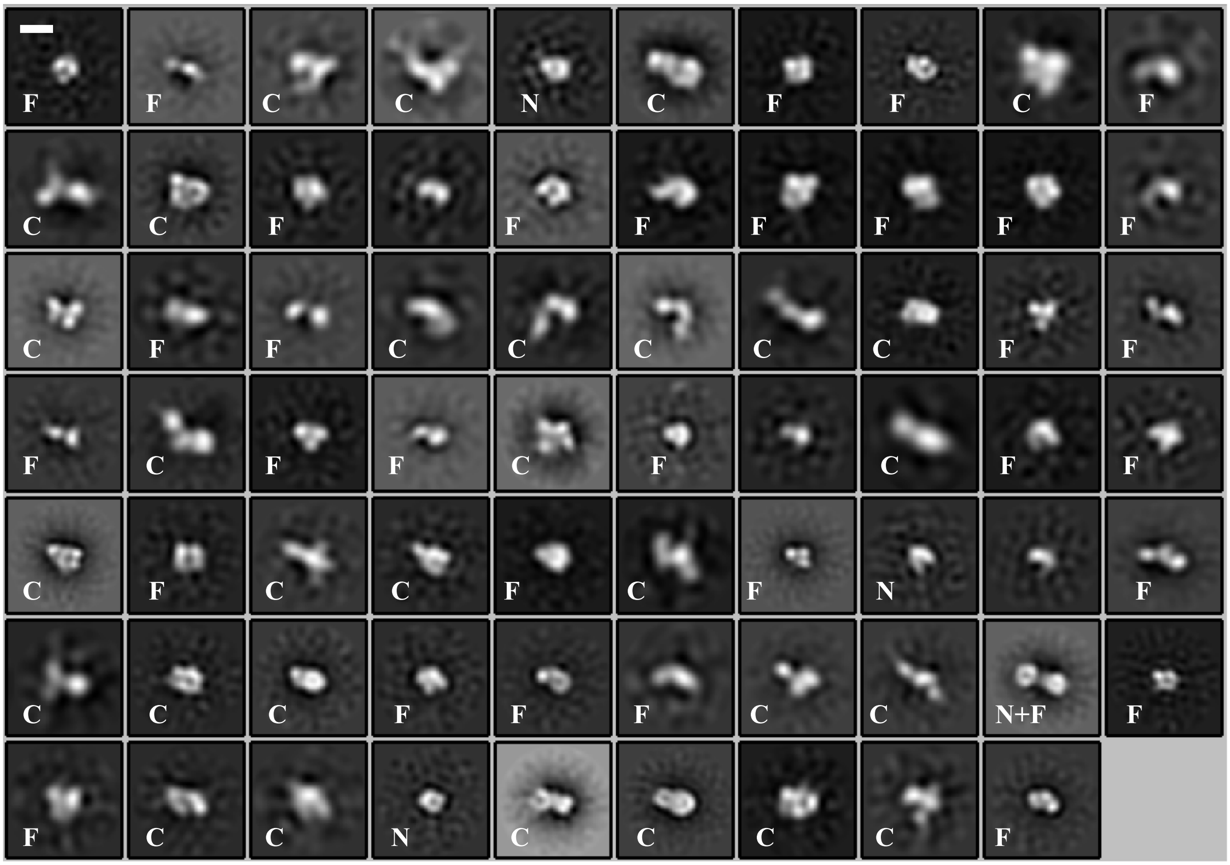

Fig. S5. 2D class-averages of FACT complexes with nucleosomes formed in the presence of curaxin CBL0137.

Nucleosome-free FACT (F), FACT-nucleosome complexes (C) and nucleosomes (N) are present in the sample. Bar – 10 nm.

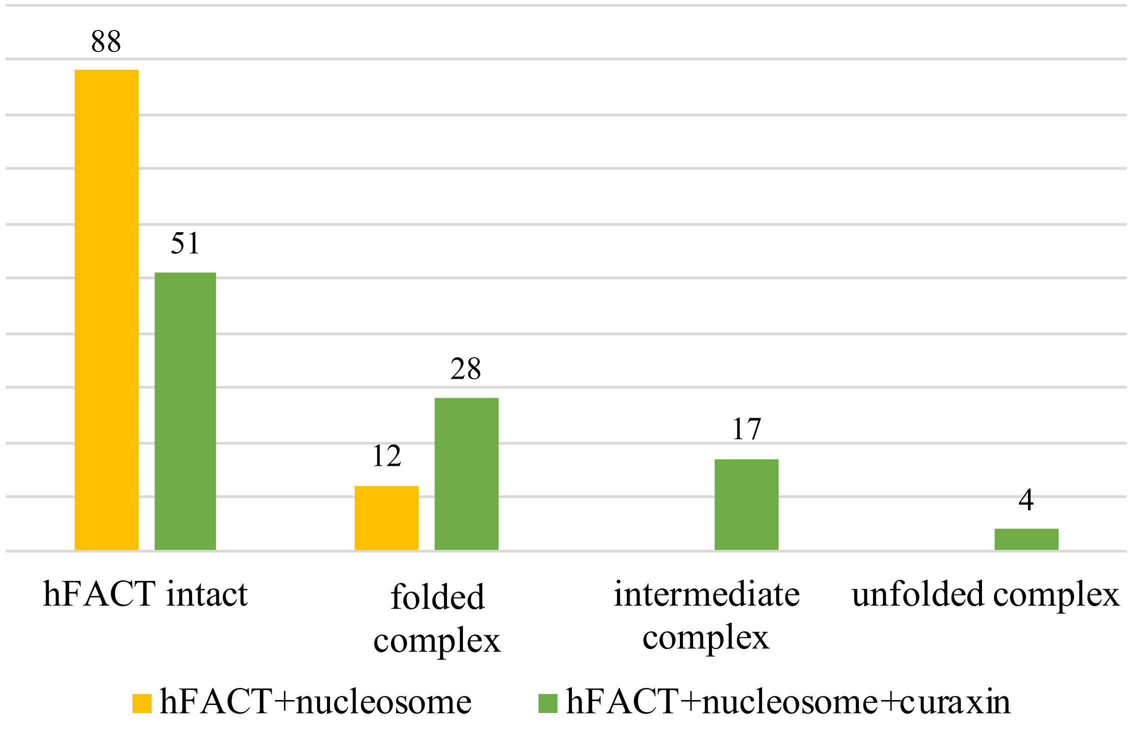

Fig. S6. Nucleosome unwrapping by FACT in the presence of curaxin CBL0137.
Fractions (%) of FACT-nucleosome complexes having different conformations in the absence (yellow) and in the presence (green) of CBL0137 are shown. Intact FACT is not bound to nucleosomes.

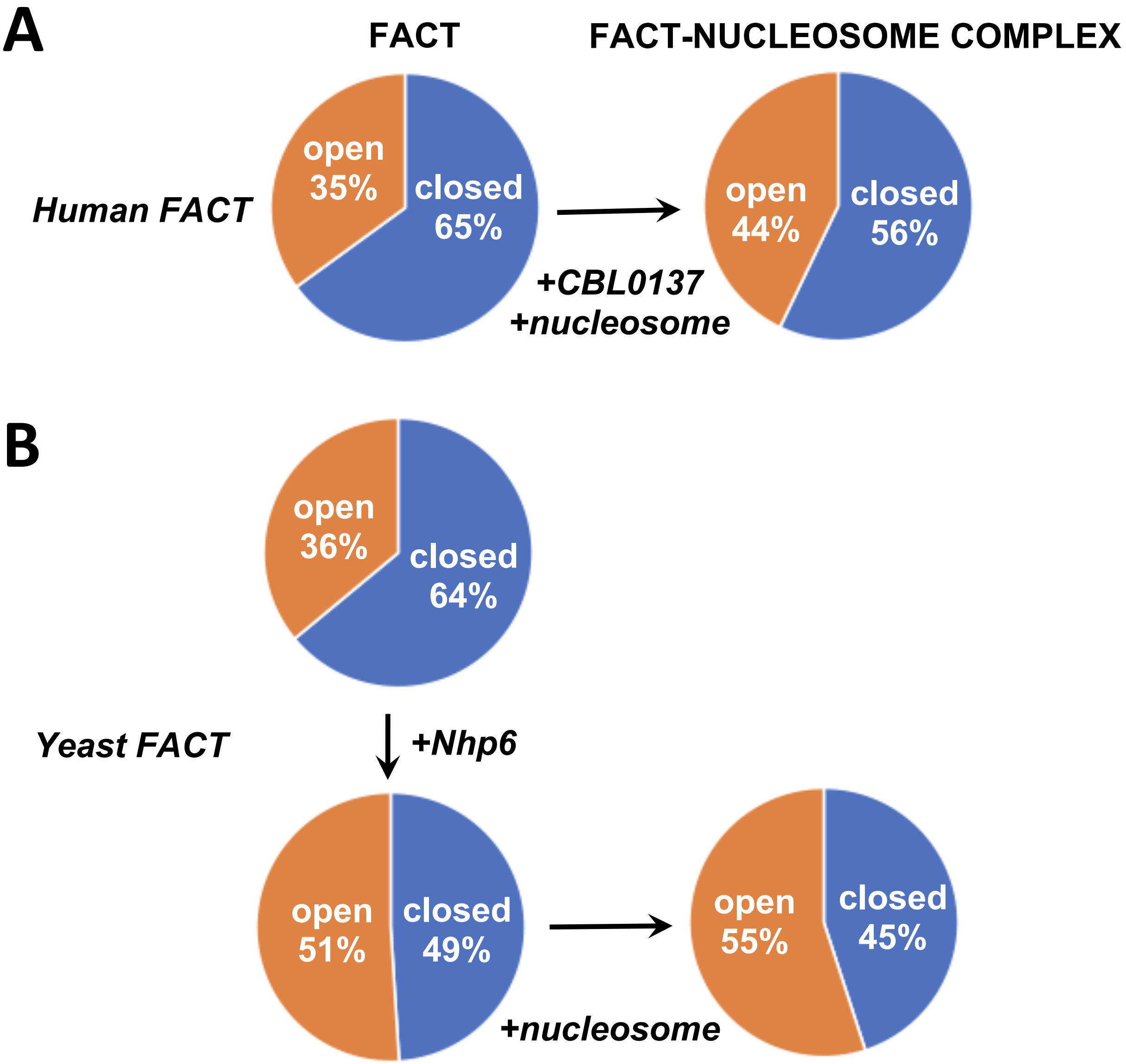

Fig. S7. Conformations of human (A) and yeast (B) FACT in solution and in FACT-nucleosome complexes.

To allow easier comparison between different samples, all complexes containing compact and open conformations of FACT were counted as closed and open complexes, respectively. **(A)** Human FACT-nucleosome complexes were formed in the presence of curaxin CBL0137. (**B)** Yeast FACT-nucleosome complexes were formed in the presence of DNA-binding protein Nhp6 that facilitates FACT opening, partial DNA uncoiling from histone octamer and greatly facilitates nucleosome unfolding (Sivkina et al., 2022).

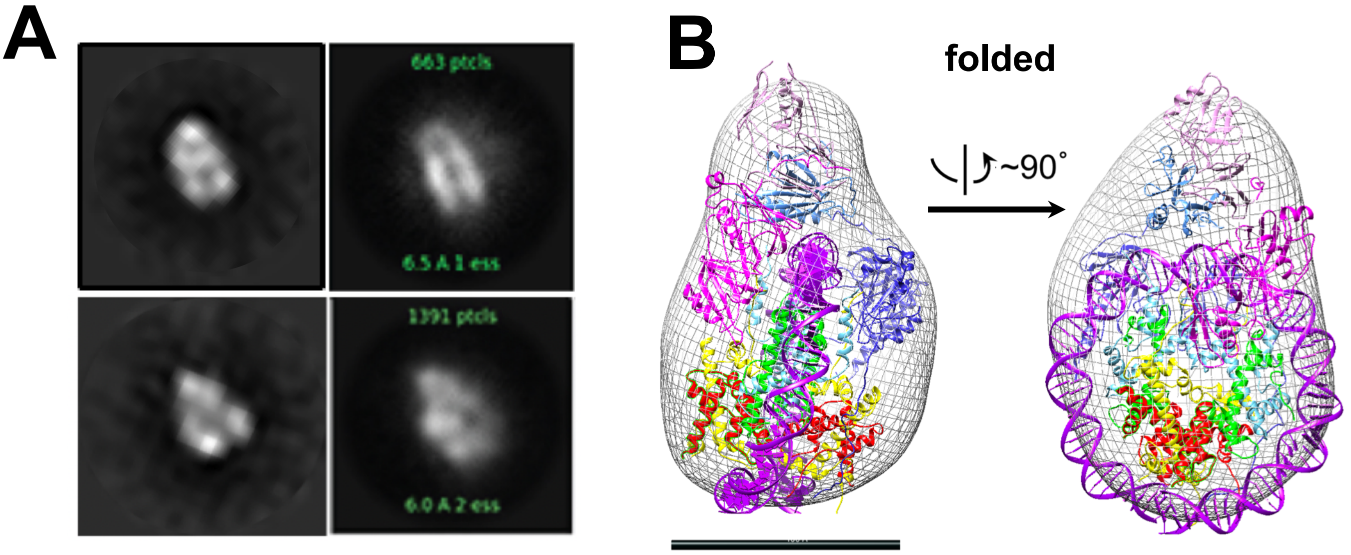

Fig. S8. Structures of folded and unfolded FACT-nucleosome complexes formed in the presence of curaxin CBL0137. (A) Comparison of similar 2D class-averages of folded FACT-nucleosome complexes from the present study (left) and from Liu et al. 2020 (right). Scale bar – 10 nm,

(**B**) A model of the compact FACT-nucleosome complex with crystal structures of SPT16-DD, SSRP1-DD, SSRP1-MD, SPT16-NTD/MD/CTD (PDB 6UPL) fitted into the electron densities of the complex with the correlation coefficient 0.92.

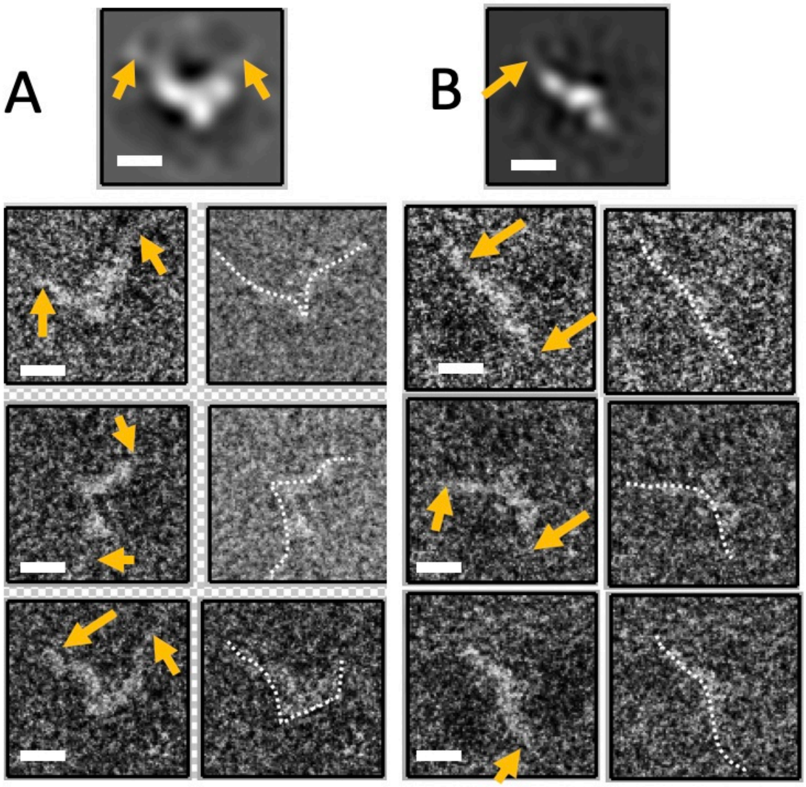

Fig. S9. DNA is retained in the FACT-nucleosome complexes unfolded in the presence of curaxin CBL0137.

Top: 2D class averages, representing: **(A)** partially unfolded complexes, **(B)** fully unfolded complexes. Below each class corresponding raw images are presented in left columns. Arrows show the flexible DNA regions. In the right columns the apparent DNA paths are depicted with white dashed lines. Bar – 10 nm. The total length of the complexes with DNA reaches 50-55 nm (about 140-150 bp), which roughly corresponds to the length of the 147-bp N35/112 DNA fragments used in this study.

Sample	Micrographs acquired	Total particles number	Number of particles used for 3D analisys
hFACT	1035	129 197	67 347
hFACT-(SPT16ΔNTD)	415	122 548	33 772
hFACT+nucleosome	1092	216 886	77 630
hFACT+nucleosome+CBL037	1965	151 846	95 634

Table S1. Numbers of particles analyzed for different samples.

Spt16 NTD E	SPT16 DD domain D	SSRP1 NTD\DD domain C	SSRP1 MD domain B
**Hydrogen bonds**			
Asp26	Tyr567<br>Tyr565<br>Asp564		
Glu43			Lys264<br>Arg269
Asp324	Lys596	Arg164	
Lys321	Glu597	Glu162	
Lys370		Glu149, Glu162	
Arg317		Glu145	
Glu311		Lys143	
Glu315	Lys596	Lys143	
Asp331	Gly576		
Gly319		Arg164	
Asn327	Ser577		
Lys335	Gly576		
Gln368	Glu562		
Cys323	Arg569		
**Hydrophobic contacts**			
Tyr170<br>Tyr369		Met161	
Val40<br>Pro143			Phe222
Pro143			Pro261<br>Ile263
Ala166	Val561		
Val167<br>Tyr28<br>Ala29<br>Tyr369	Tyr565		
Ala166<br>Val167<br>Tyr170<br>Tyr369	Tyr567		

Table S2. Amino acids participating in interactions between Spt16 NTD and other domains of human FACT subunits. Residues that are identical or similar in properties between yeast and human FACT are shown in pink.
